# Pre-treatment high body mass index is associated with poor survival in Asian premenopausal women with localized breast cancer

**DOI:** 10.7150/jca.59133

**Published:** 2021-05-27

**Authors:** Yung-Chang Lin, Hsiao-Hsiang Cheng, Shin-Cheh Chen, Wen-Chi Shen, Yi-Ting Huang

**Affiliations:** 1 Division of Hematology/Oncology, Department of Internal Medicine, Chang Gung Memorial Hospital, Linkou Branch, Tao-Yuan, Taiwan.; 2 Kou Foundation Sun Yat-Sen Cancer Center, Taipei, Taiwan.; 3 School of Medicine, Chang Gung University, Tao-Yuan, Taiwan.; 4 Department of General Surgery, Chang Gung Memorial Hospital, Linkou Branch, Tao-Yuan, Taiwan.; 5 Department of Radiotherapy, Chang Gung Memorial Hospital, Linkou Branch, Tao-Yuan, Taiwan.

**Keywords:** breast cancer, obesity, prognostic factor, body mass index

## Abstract

**Background:** Obesity is associated with poor prognosis in breast cancer patients. This study aimed to evaluate the effect of obesity measured by body mass index (BMI) on survival of Taiwanese breast cancer patients in a single institution.

**Methods:** We observed 5000 patients who were diagnosed with stage I-III breast cancer between 1990 and 2005. Information on BMI at diagnosis, and clinical follow-up for disease recurrence and death, up to 20 years post-diagnosis were available. BMI (in kg/m^2^) categories included normal weight (BMI<24), overweight (24≤BMI<27), and obesity (BMI≥27), according to recommendations from the Bureau of Health Promotion of Taiwan. The role of BMI and other known prognostic factors for patient survival were evaluated in this patient cohort.

**Results:** Obesity was associated with advanced stage, higher nuclear grade, and higher percentages of estrogen receptor (ER) positive. The median age of patients with a higher BMI was greater than the median age of patients with a lower BMI. Obesity was an independent prognostic factor of overall survival (OS) (*P*<0.001), but not disease-free survival (DFS) (*P*=0.067). We subsequently analyzed the impact of age-stratified BMI (age<50 and age≥50 years) to ameliorate the impact of age bias. Following subset analyses, obesity correlated with shorter DFS (*P*=0.004) and OS (*P*=0.009) only in women<50 years of age**.** Multivariate analysis revealed that BMI was an independent prognostic factor for both DFS and OS in this group of patients. Subset analysis revealed that in women <50 years old, the impact of BMI on survival was associated with higher stage, ER negativity.

**Conclusion:** BMI is an independent prognostic factor of OS and DFS in breast cancer patients aged<50 years. Although the cause-effect relationship between obesity and survival is unclear, we recommend that weight control measures in young breast cancer survivors should be considered.

## Introduction

Obesity is an emerging public health issue worldwide 1. It is linked not only with type 2 diabetic mellitus and cardiovascular disease, but also with an increased risk of developing several cancer types 2, 3. Obesity is an established risk factor for breast cancer, and is also associated with an increased risk of endometrial, kidney, colon, and esophageal cancer 4-6.

Sex hormone-associated cancers such as breast cancer are related to, at least in part, an elevated level of endogenous hormones 7. Obesity may promote breast cancer progression through various mechanisms, including increased sex hormone levels in post-menopausal obese women 8-10. Interestingly, meta-analyses have shown that obesity increases the risk of breast cancer in postmenopausal women, but decreases the risk in premenopausal women 8, 11. This suggests that the effect of obesity on breast cancer biology might vary in different contexts of hormonal status, hormonal therapy, or other unknown mechanisms 5, 12-16. A meta-analysis focus on premenopausal from Asian patients found that obesity is associated with breast cancer, whether the result is associated with ethic difference was unknown 17. In breast cancer, obesity also has been shown to be associated with poor overall survival (OS) and disease-free survival (DFS) 18-23, advanced stage at diagnosis 24, unfavorable tumor grade, and suboptimal local and systemic treatment results 19-21, 25, 26. Obesity could affect chemotherapy efficacy, and is reported to be an independent prognostic factor for pathological complete response rate after neoadjuvant chemotherapy 27. However, these studies are inconsistent. Some studies show that body mass index (BMI) is an independent factor affecting OS or DFS in breast cancer patients 15, 19, 20, 23, 25, 28-31, while some show that obesity is prognostic factor only in premenopausal women 23, 31, 32. A meta-analysis found that the effect of obesity was greater in premenopausal women than in postmenopausal women, although the hazard ratio was statistically insignificant 20. Inconsistencies in determining the effect of obesity on breast cancer prognosis might be attributable to several factors such as the study methodology, patient size and population, and duration of follow-up. Patient ethnicity might also be taken into consideration.

The prevalence of obesity is relatively lower in Asian women than in Caucasian women. The definition of obesity in the Taiwanese population is BMI of ≥27 as most Taiwanese studies adopted 33, 34, rather than the commonly used cutoff value of 30 in Western countries 18. This was based on a national nutrition and health survey conducted between 1993-1996 35, it showed that for most BMI values, the prevalence of metabolic disorders were higher for Taiwanese than for Caucasians, and BMI 27.5 were the cutoffs with the highest predictive value 36. A few studies have suggested that obesity, despite its low prevalence in Asian women, remains a risk factor of developing breast cancer 37. In this work, we retrieved the data from institutional cancer registry since 1990, and medical records review. The diagnoses and management of the patients in this cohort were, generally, in accordance with the common worldwide guidelines of standards such as the National Comprehensive Cancer Network or St. Gallen consensus 38. This allows us to analyze the effect of BMI on survival of Taiwanese breast cancer patients in a practice-oriented setting. Herein, we report the results of the analysis of the effect of BMI on survival of Taiwanese breast cancer patients, and the relationships between BMI and known prognostic factors.

## Patients and Methods

Between 1990 and 2005, a total of 5000 female breast cancer patients who underwent breast cancer surgery were registered at a single institution. Information on patient demographics, tumor characteristics, and adjuvant or neo-adjuvant chemotherapy, radiotherapy, or hormonal therapy and disease status was collected retrospectively from medical records. Information regarding BMI at the time of diagnosis was available. Patients were followed up for recurrence and death for up to 20 years. The dates of death were confirmed by national vital statistics data provided by the Taiwanese Department of Health. The conduct of this study was in accordance of declaration of Helsinki and was approved by the Institutional Review Board.

### Statistical analysis

Patient and tumor characteristics within different BMI subsets were analyzed. Continuous variables were analyzed with repeated measures of analysis of variance, and categorical variables were analyzed by the chi-square or Fisher's exact test. DFS was calculated from the date of primary surgery to the date of documented recurrence. Patients who died, or were lost to follow-up without documented evidence of freedom from recurrence, were censored at the date of death or the last patient visit. OS was calculated from the date of primary surgery to the date of death.

The OS and DFS were estimated according to the Kaplan-Meier method. Differences in survival rates were calculated using the log-rank test. Prognostic factors including: tumor size, nodal status, hormone receptor status, and Human Epidermal Growth Factor Receptor 2 (HER2) status, were analyzed according to the Cox regression model for OS and DFS. A *P* value of <0.05 was considered significant. All statistics were calculated using the SPSS software package (SSPS Inc., Chicago, IL, USA).

## Results

Between 1990 and 2005, data from 5000 female breast cancer patients were compiled. Patient BMI data were obtained at diagnosis and ranged from 13.5 to 48.3 (mean = 24.26). The definitions of obesity and other categories were based on BMI according to recommendations from the Bureau of Health Promotion of Taiwan: normal weight, BMI <24; overweight, 24≤ BMI <27; and obesity, BMI ≥27. The numbers of normal weight, overweight and obese patients were 2605 (52.1%), 1300 (26%), and 1095 (21.9%), respectively. The median age (years) for the three groups was 44.7, 49.8, and 52.5, respectively (*P*<0.001). Despite the BMI definition of obesity being much lower than the commonly used BMI criteria in Western countries, obese Taiwanese patients had larger tumors (median size: 2.78 cm, *P*<0.001) and more advanced nodal statuses (*P* =0.007), compared to normal weight and overweight patients. Tumors in obese women were associated with higher nuclear grades (*P* <0.001) and higher percentages of positive ER (*P*=0.011) and negative HER2 expression (*P*=0.039). In summary, the clinic-pathological features in obese breast cancer patients were mixed, with both favorable and unfavorable factors. The treatments that patients received, however, did not differ between the leaner and heavier breast cancer patients. In order to eliminate any potential effect of patient age on these factors, we arbitrarily grouped this cohort into patients <50 and ≥ 50 years old, to represent pre- and post-menopause status respectively. There was no accurate information regarding the patients' menopausal status at diagnosis. The patient demographics and tumor characteristics, divided by age group and BMI category, are summarized in Table 1. The distribution pattern of these parameters was similar between the younger and older age groups. We found that nodal stage and nuclear grade were statistically more favorable in leaner patients within the premenopausal group. In post-menopausal women, a higher percentage of obese patients had hormonal receptor positive tumors; but there was no association between HER2 expression and BMI categories of either pre- or post-menopausal patients.

**Figure 1:**
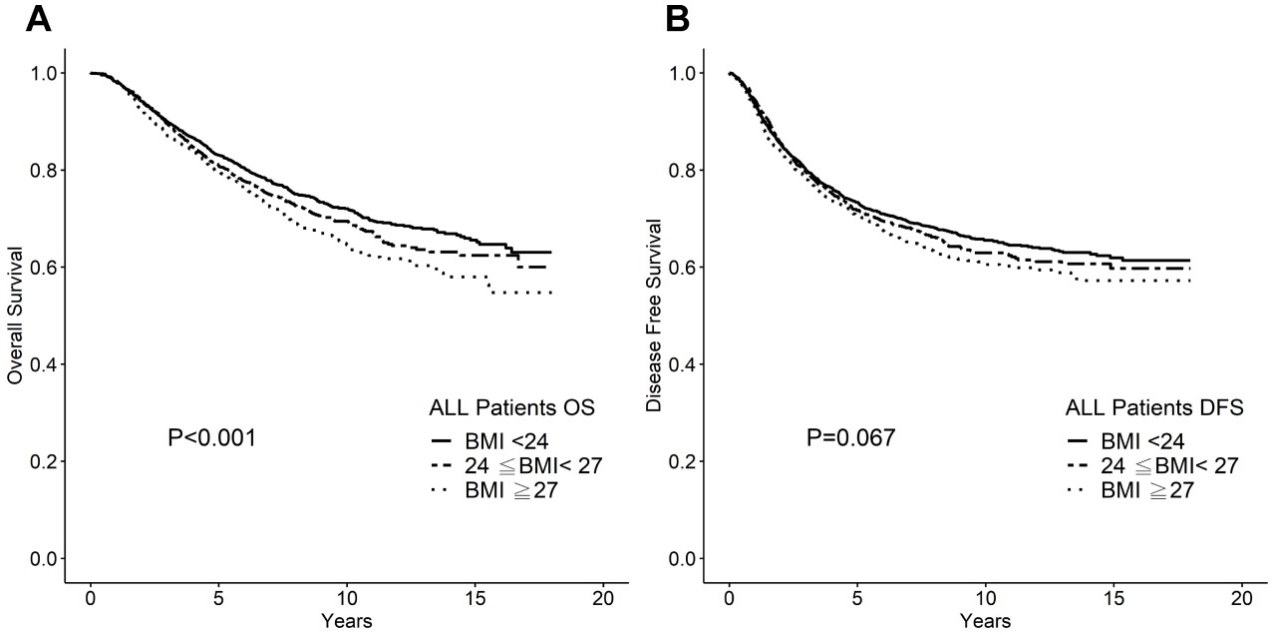
** A.** Overall survival according to BMI category for whole cohort. **B.** Disease-free survival according to BMI category for whole cohort.

Higher BMI significantly associated with poor OS but not with DFS in a uni-variate analysis, as shown in Figure 1. The 10-year OS rates were 72%, 69% and 66% for the normal weight, overweight, and obese BMI groups, respectively (*P* <0.001). The 10-year DFS rates were 66%, 65%, and 63% for the normal weight, overweight, and obese groups, respectively (*P* =0.067). When used BMI 30 as cutoff, the number of obese patients was only 433 (8.66%), and the difference of 10-year DFS and OS were insignificant (p= 0.289, and 0.110, respectively). Even after multivariate analysis with the other known prognostic factors including tumor stage (T stage), nodal stage, ER status, and HER2 status, BMI remained an independent prognostic factor for OS (*P* =0.007; Table 2). Owing to our data retrieval limitations, we did not conduct a breast cancer-specific survival analysis. It is possible that the effect of BMI on OS could be attributed to co-morbidity or simply to age-related factors.

**Figure 2:**
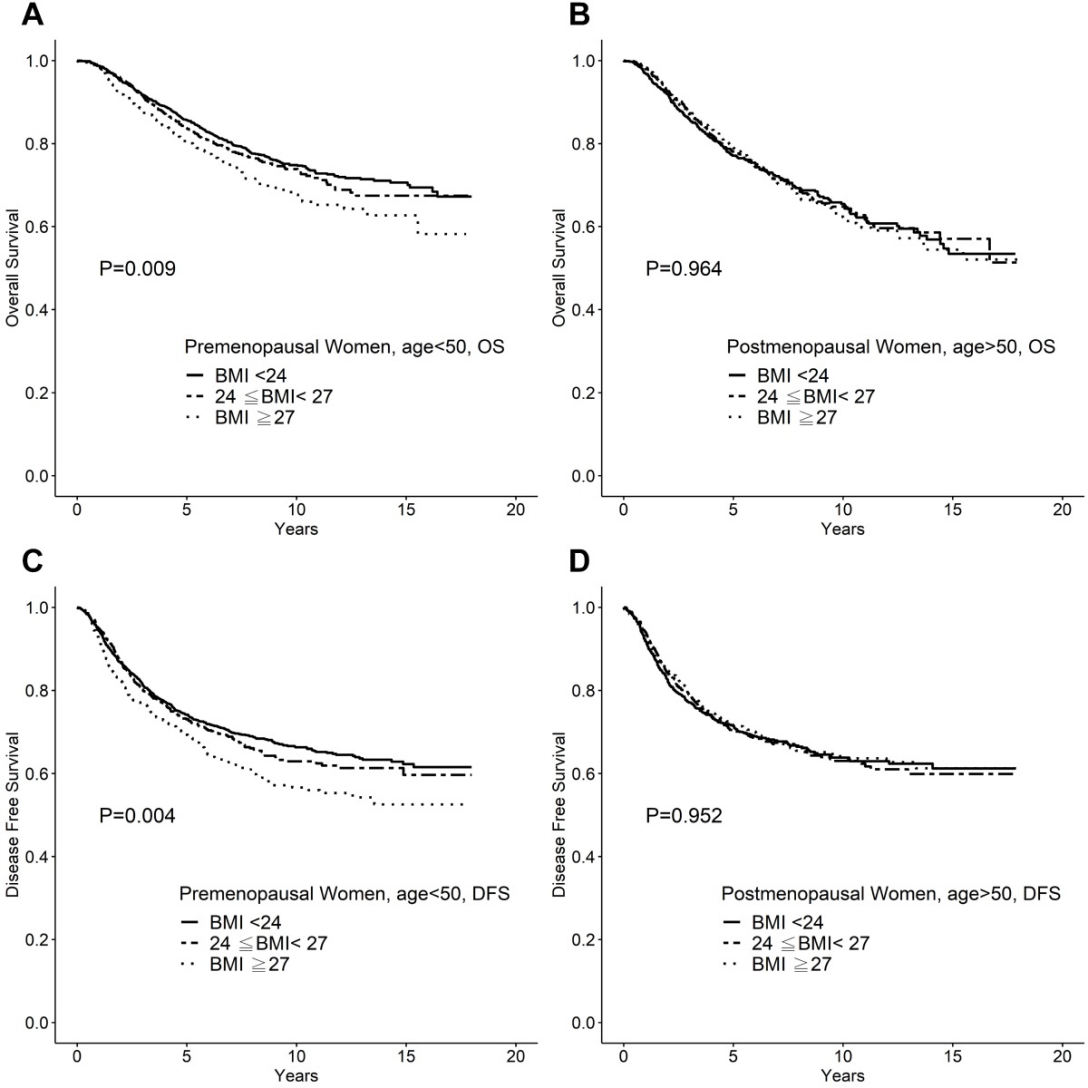
** A.** Overall survival of breast cancer patients <50 years of age. **B.** Overall survival of breast cancer patients aged ≥50 years. **C.** Disease-free survival of breast cancer patients <50 years of age. **D.** Disease-free survival of breast cancer patients aged ≥50 years.

We further tested whether menopause status affects BMI as a prognostic factor, we analyzed the impact of BMI on survival by stratifying the patients into two age groups: <50 years and ≥50 years. The clinicpathological features were compared between the two patient groups. The younger patient group was associated with smaller tumors, lower nodal status, and low nuclear grades. In breast cancer patients aged <50 years, the 10-year OS rates were 75%, 73%, and 69% (*P* = 0.009), and the 10-year DFS rates were 67%, 65%, and 60% (*P* =0.004), for normal weight (BMI <24), overweight (24≤ BMI <27), and obese patients (BMI ≥27), respectively. In contrast, for breast cancer patients aged ≥50 years, neither the OS (*P*=0.964) nor DFS (*P* =0.965) were affected by BMI (Figure 2).

The significance of BMI as a prognostic factor for patients <50 years old was further demonstrated by multivariate analysis. Known prognostic factors such as tumor stage, nodal stage, as well as ER status along with HER2 status were selected for analysis in this cohort. The result indicated that BMI, T stage, nodal stage, ER status, and nuclear grade were independent prognostic factors for both DFS and OS (Table 3).

We subsequently analyzed the interactions between BMI and other established prognostic factors. Table 4 demonstrates the differences in 10-year survival rates for each T stage, nodal stage, and ER or HER2 status, stratified by BMI. For patients <50 years of age, the impact of BMI on survival was more significant in the node-positive and ER-negative patients (OS: *P =*0.045 and 0.001, respectively; DFS: *P* =0.049 and 0.001, respectively).

The prognostic value of intrinsic subtypes is an interesting issue to be studied. We sorted 2758 patients whose HER2 data was available and classified into hormone receptor (HR) positive/HER2 negative (n=1196); HER2 positive (either HER2 IHC 3+ or FISH positive) (n=769); and triple negative (TNBC) (n=793) subtypes. We compared the difference of survivals using BMI 27 as cutoff on each subtypes. We found that BMI ≥27 remained a statistically significant factor for poor disease free survival (p=0.01) and overall survivals for TNBC (p=0.009) breast cancer patients < 50 years of age, but not elder patients (Table 5).

## Discussion

The present study reveal that BMI at diagnosis, is an independent prognostic factor for survival in premenopausal Asian breast cancer patients. We found that higher-BMI patients present mostly with larger tumor sizes, advanced stage, higher nuclear grades, and higher percentages of ER positivity. A BMI ≥27 is statistically associated with poor OS but doesn't significantly affect DFS. The impact of BMI on OS affects only on patients aged <50 but not ≥50 years old. In patients aged <50 years, a BMI ≥27 is an independent prognostic factor for both DFS and OS, after adjusting for tumor size, nodal status, ER status, and HER2 status. Our study suggests that the effect of obesity is context-dependent with regard to age, and menopausal status.

We and other studies have shown that obese breast cancer patients tend to have an advanced stage, higher nuclear grade, but more HR positive tumors 18, 24, 26. The plausible mechanisms for the association of high tumor size, stage; and nuclear grade with obese breast cancer patients were multi-factorial 24. One of the explanations for the association with advanced stage, or tumor size was the delayed diagnosis due to obesity 26. Biological mechanism such as the insulin resistance/insulin like growth factor-1 axis, enriched humoral factors such as adipokines, pro-inflammatory cytokines have been addressed 39-41. The reports on the association of breast cancer subtypes with obesity were inconsistent. Majority of the reports did not find a positive correlation with any subtypes of breast cancer with obesity, whereas some reported increased ER positive cancers on obese patients 26, 28. As estrogen is a promotor for breast cancer progression, our data showed that upon subgroup analysis, the association of increased ER positive breast cancers was only on the post-menopausal subgroup. This feature suggests that increased estrogen level on post-menopausal obese women is a possible explanation. Our whole cohort analysis suggested that HER2 negativity had a marginal positive correlation with higher BMI. However, the association did not exist on the subsequent subset analysis, regardless the age, or BMI categories. Therefore, the association of HER2 negativity with obesity on our dataset was inconclusive.

A meta-analysis conducted by Protani et al. analyzed 43 relevant studies published between 1960 and 1990, in which obesity was measured by either BMI or waist-hip ratios. The conclusion was that obesity was modestly associated with poor overall and breast cancer-specific survival 20. However, the study period spanning over three decades, the reported studies might not have the same quality of treatments. Jiralerspong et al in another metanalysis, using more updated data, also concluded similar results. 42 Several large scale population-based studies have been addressed the influence of obesity on both OS and DFS. Majed et al. used a BMI of 30 as a cutoff value and followed a breast cancer cohort for up to 20 years. The authors found that obesity was associated with shorter distant metastasis-free and secondary malignancy-free times. Disease-free intervals and survival were reduced among obese patients 21. A Danish study enrolled more than 50,000 patients who were diagnosed between 1977 and 2006; BMI information was available for only approximately 1,900 patients who received chemotherapy. That could have a bias toward advanced, and HR negative population. The times to distant metastasis and breast cancer-related death, were significantly worse among patients with BMI >30, compared to those with BMI <25 19. Ligibel et al. did a post hoc analysis on CALGB 9741 study and found that BMI is an independent prognostic factors for survivals on patients receiving optimal dose adjuvant chemotherapy 43. In summary, in unselected population or chemotherapy-treated population, obesity was a prognostic factors for breast cancer survivals.

The differently weighted values of obesity with regard to menopausal status have been addressed as well. Loi et al. reviewed 10 different studies on premenopausal or postmenopausal patients with varied BMI category definitions. Although they concluded that obesity was an independent prognostic factors for both groups, the HR ratio was even greater on the pre-menopausal group 31. Whiteman divided approximately 2,500 breast cancer patients into four groups according to BMI, and found a significant trend toward an increased death rate in obese peri- and premenopausal women, but not in obese postmenopausal women 23. Berclaz et al. analyzed an International Breast Cancer Study Group (IBSCG) trial cohort of patients and showed that decreases in DFS and OS among obese (BMI>30) patients were limited to pre- and perimenopausal patients 32. Study from Protani et al also showed similar trends that the effect of obesity on survival was more significant in premenopausal women 20. Kim, et al., however did an analysis from Korean on stage I-III patients and concluded a contradictory results that obesity was associated with negative impact of relapse free survival only in HR positive and postmenopausal groups 44.

The inconsistent data derived from these studies might be due to relatively small sample sizes or inadequate follow-up periods. Our study cohort was relative large (N=5,000) and was the longest follow-up period up to 20 years. The results generated from our study are comparable with the majority of the studies that suggest that obese women with poor survival rates are mainly premenopausal (age <50 years) women. The dichotomy of the effect of obesity on breast cancer is interesting. Obesity increases the breast cancer risk in postmenopausal women, but is inversely related to the risk in premenopausal women 45. On the basis of the results from our study and others, obesity seems to be associated with poor prognosis in premenopausal women, but not in postmenopausal women. To date, the underlying mechanism of this finding is unclear, and mostly hypothetical 41, 46, 47. Since breast cancer is a sex hormone-associated malignancy, the role of sex hormones has been discussed at length, even in the Chinese population 48. Possible explanations included: advanced stage at diagnosis, increased paracrine sex hormones in the surrounding adipose tissue 49, attenuated post-chemotherapy amenorrhea effects in obese women, and lower levels of circulating estradiol in premenopausal women who were more sensitive to high-ER tumor status. Another hypothesis for the higher risk of recurrence in obese premenopausal women is that non-sex hormonal mechanisms might play a role 39, 50. Our subgroup analysis showed that the effect of obesity on prognosis was more significant in ER-negative patients or TNBC subset, suggesting that an ER-independent pathway might be involved. For example, leptin, a growth factor for malignant breast cells, might play a role in the mechanism underlying the association between BMI and breast cancer 40, 51. Obesity might be associated with a untoward immune microenvironment for host immunity against breast cancer 52. However, no causal relationships have been confirmed. We also noted that obese patients were increased rate of positive node status, and would increase recurrence and mortality. According to our treatment guidelines, the group of patients should receive adjuvant chemotherapy and radiotherapy if they present with pathological T3 or N2 disease. The GEICAM study group analyzed pooled data from three different adjuvant chemotherapy clinical trials and found that severe obesity is a poor prognostic factor across all subtypes of breast cancers 53. A Mexican study recently found that obesity is an independent poor prognostic factor for locally advanced breast cancer receiving neo-adjuvant chemotherapy 54. The causal relationship is unknown; whether it could be attributed to suboptimal chemotherapy for obese patients is not clear.

The limitation of our study is that these data were collected from a single institute and, thus, might not adequately represent the overall Asian population. The study lacks cancer-specific survival data, and there was no difference in disease-free survival on whole cohort, which might be biased by confounding factors. However, since Asian women tend to be much thinner than Caucasian women, a cutoff BMI value of 27 could be applicable for this population 33, 35, 55. In contrast to population-based studies, our patients received guideline-oriented therapy and regular follow-up at single institution. Our patients included all stage I to III breast cancer patients who received primary surgery, regardless of the presence or absence of subsequent adjuvant therapy; therefore, this information is more close to real world data than that of clinical trial-based cohorts. The data generated for this analysis are relatively reliable. Although there is lack of cause-specific survival, the significance of obesity on DFS and OS was confirmed by a multivariate analysis.

In summary, we confirmed that in an Asian population, young obese women had a higher risk of breast cancer recurrence and mortality than lean women. This tendency was more prominent in patients with ER-negative and advanced-stage tumors. We suggest that further investigations of the underlying mechanism and strategies to improve outcomes in this population are warranted.

## Figures and Tables

**Table 1:** Demographic of Patients and Tumor Characteristics (N=5000)

Characteristic		BMI (kg/m^2^)							
		<24 (n = 2605)		24≤ BMI <27 (n = 1300)			≥27 (n = 1095)		
Median age (mean)	*P* <0.001	44 (46)		49 (51)			52 (53)		
		Age <50 (n = 2923)				Age ≥50 (n = 2077)			
		***BMI (kg/m^2^)***				***BMI (kg/m^2^)***			
		***<24***	***24≤BMI <27***		***≥27***	***<24***		***24≤BMI<27***	***≥27***
	No. (%)	1809 (61.9)	661 (22.6)		453 (15.5)	796 (38.3)		639 (30.8)	642 (30.9)
Mean tumor size (cm)		2.5	2.57		2.89	2.48		2.56	2.70
	*P* value	0.043*	0.143**			0.024*		0.662**	
T (%)	T1	748 (41.93)	272 (41.1)		154 (34)	296 (37.2)		243 (38.1)	221 (34.4)
	T2	910 (50.3)	335 (50.7)		256 (56.6)	446 (56.0)		349 (54.6)	369 (57.5)
	T3	118 (6.5)	46 (7.0)		35 (7.7)	41 (5.2)		32 (5.0)	37 (5.8)
	T4	27 (1.5)	7 (1.1)		8 (1.8)	13 (1.6)		14 (2.2)	14 (2.2)
	Unknown	6 (0.3)	1 (0.2)		0	0		1 (0.2)	1 (0.2)
	*P* value	0.056*	0.135**			0.683*		0.819**	
N (%)	N0	1008 (55.7)	364 (55.1)		228 (50.3)	446 (56.0)		328 (51.3)	330 ( 1.4)
	N1	432 (23.9)	158 (23.9)		100 (22.1)	155 (19.5)		144 (22.5)	137 (21.3)
	N2	185 (10.2)	77 (11.6)		66 (14.6)	85 (10.7)		91 (14.2)	82( 12.8)
	N3	173 (9.6)	60 (9.1)		56 (12.4)	100 (12.6)		73 (11.4)	88 (13.7)
	Unknown	11 (0.6)	2 (0.3)		3 (0.7)	10 (1.3)		3 (0.5)	5 (0.8)
	*P* value	0.022*	0.143**			0.343*		0.638**	
ER status (%)	Negative	939 (51.9)	361 (54.6)		237 (52.3)	440 (55.3)		331 (51.8)	313 (48.8)
	Positive	659 (36.4)	241 (36.5)		178 (39.3)	286 (35.9)		254 (39.7)	275 (42.8)
	Unknown	211 (11.7)	59 (8.9)		38 (8.4)	70 (8.8)		54 (8.5)	54 (8.4)
	*P* value	0.543*	0.363**			0.007*		0.249**	
PR status (%)	Negative	935 (51.7)	344 (52.0)		234 (51.7)	476 (59.8)		364 (57.0)	361 (56.2)
	Positive	659 (36.4)	256 (38.7)		179 (39.5)	247 (31.0)		221 (34.6)	227 (35.4)
	Unknown	215 (11.9)	61 (9.2)		40 (8.8)	73 (9.2)		54 (8.5)	54 (8.4)
	*P* value	0.463*	0.831**			0.096*		0.771**	
NBR (%)	1	223 (12.3)	71 (10.7)		67 (14.8)	92 (11.6)		70 (11.0)	89 (13.9)
	2, 3	802 (44.3)	321 (48.6)		241 (53.2)	389 (48.9)		341 (53.4)	354 (55.2)
	Unknown	784 (43.3)	269 (40.7)		145 (32.0)	315 (39.6)		228 (35.7)	199 (31.0)
	*P* value	<0.0001*	0.015**			<0.0001*		0.212**	
HER2 status (%)	Negative	666 (36.8)	262 (39.6)		181 (40.0)	288 (36.2)		245 (38.3)	246 (38.3)
	Positive	251 (13.9)	70 (10.6)		63 (13.9)	125 (15.7)		96 (15.0)	97 (15.1)
	Unknown	892 (49.3)	329 (49.8)		210 (47.1)	383 (48.1)		298 (46.6)	299 (46.6)
	*P* value	0.628*	0.183**			0.550*		0.970**	
Adjuvant chemotherapy (%)	No	336 (18.6)	118 (17.9)		59 (13.0)	254 (31.9)		216 (33.8)	206( 32.1)
	Yes	1473 (81.4)	543 (82.1)		394 (87.0)	542 (68.1)		423 (66.2)	436 (67.9)
	*P* value	0.005*	0.030**			0.943*		0.514**	
Hormonal therapy (%)	No	1043 (57.7)	393 (59.5)		260 (57.4)	395 (49.6)		303 (47.4)	303( 47.2)
	Yes	694 (38.4)	243 (36.8)		178 (39.3)	371 (46.6)		323 (50.5)	314( 48.9)
	Unknown	72 (4.0)	25 (3.8)		15 (3.3)	30 (3.8)		13 (2.0)	25 (3.9)
	*P* value	0.776*	0.665**			0.657*		0.142**	
									

Abbreviations: NBR: nuclear grade; *P value for the comparison of the BMI <24 group and BMI ≥27 group; **P value for the comparison of the 24≤BMI<27 group and BMI ≥27 group.

**Table 2:** Multivariate Cox Regression Analysis for the OS and DFS of breast cancer patients

	DFS			OS		
	Hazards Ratio	95% CI	*P* value	Hazards Ratio	95% CI	*P* value
All patients			0.569			0.015
BMI <24	1.000			1.000		
24≤ BMI <27	1.019	0.910-1.142	0.740	1.136	1.011-1.277	0.033
BMI ≥27	1.046	0.929-1.178	0.454	1.174	1.039-1.326	0.010
**Tumor size**			<0.0001			<0.0001
T1	1.000			1.000		
T2	1.402	1.254-1.567	<0.0001	1.515	1.347-1.705	<0.0001
T3	2.011	1.681-2.406	<0.0001	2.410	2.011-2.889	<0.0001
T4	4.151	3.177-5.425	<0.0001	4.881	3.749-6.354	<0.0001
**Nodal status**			<0.0001			<0.0001
N0	1.000			1.000		
N1	2.436	2.148-2.763	<0.0001	2.057	1.802-2.348	<0.0001
N2	3.569	3.100-4.109	<0.0001	3.224	2.791-3.724	<0.0001
N3	5.818	5.067-6.681	<0.0001	5.422	4.712-6.239	<0.0001
**NBR**			0.001			<0.0001
1	1.000			1.000		
2,3	1.350	1.130-1.613	0.001	1.345	1.117-1.620	0.002
Unknown	1.159	0.964-1.394	0.117	1.103	0.910-1.337	0.319
**ER status**			<0.0001			<0.0001
Positive	1.000			1.000		
Negative	1.268	1.143-1.407	<0.0001	1.295	1.162-1.443	<0.0001
Unknown	1.314	1.096-1.576	0.003	1.311	1.093-1.573	0.003
**HER2 status**			0.018			<0.0001
Negative	1.000			1.000		
Positive	1.137	0.983-1.315	0.083	1.273	1.094-1.482	0.002
Unknown	1.167	1.045-1.304	0.006	1.303	1.161-1.462	<0.0001

**Table 3:** Multivariate Cox Regression Analysis for OS and DFS of Breast Cancer Patients <50Years of Age

	DFS			OS		
	Hazards Ratio	95% CI	*P* value	Hazards Ratio	95% CI	*P* value
**All patients**			0.038			0.048
BMI <24	1.000			1.000		
24≤ BMI <27	1.063	0.913-1.237	0.432	1.175	0.996-1.386	0.056
BMI ≥27	1.239	1.051-1.460	0.011	1.208	1.004-1.453	0.045
**Tumor size**			<0.0001			<0.0001
T1	1.000			1.000		
T2	1.370	1.187-1.581	<0.0001	1.648	1.393-1.949	<0.0001
T3	1.987	1.587-2.488	<0.0001	2.779	2.181-3.539	<0.0001
T4	3.759	2.579-5.480	<0.0001	4.566	3.099-6.726	<0.0001
**Nodal status**			<0.0001			<0.0001
N0	1.000			1.000		
N1	2.290	1.951-2.688	<0.0001	2.181	1.816-2.619	<0.0001
N2	3.341	2.780-4.014	<0.0001	3.431	2.804-4.199	<0.0001
N3	4.904	4.073-5.904	<0.0001	5.001	4.087-6.120	<0.0001
**NBR**			<0.0001			<0.0001
1	1.000			1.000		
2,3	1.477	1.176-1.856	0.001	1.703	1.300-2.231	<0.0001
Unknown	1.187	0.938-1.503	1.531	1.234	0.933-1.632	0.141
**ER status**			0.001			<0.0001
Positive	1.000			1.000		
Negative	1.258	1.098-1.441	0.001	1.438	1.233-1.678	<0.0001
Unknown	1.389	1.105-1.747	0.005	1.505	1.170-1.935	0.001
**HER2 status**			0.207			0.003
Negative	1.000			1.000		
Positive	1.075	0.886-1.305	0.464	1.184	0.953-1.469	0.127
Unknown	1.138	0.986-1.314	0.076	1.323	1.125-1.555	0.001

**Table 4:** Ten-year Disease Free Survival rate and Overall Survival rate of breast cancer patients <50 years of age

	DFS (%)				OS (%)			
	BMI (kg/m^2^)				BMI (kg/m^2^)			
	<24	24≤ BMI <27	≥27	*P* value	<24	24≤ BMI <27	≥27	*P* value
**Tumor size**								
T0-1	77.1	71.4	59.9	0.222	86.6	82.9	83.2	0.371
T2-4	59.9	59.2	54.1	0.071	62.7	66.1	62.0	0.058
**Nodal status**								
Negative	81.2	79.6	77.5	0.361	86.0	85.2	84.2	0.633
Positive	50.1	47.4	41.7	0.049	61.9	58.0	53.8	0.045
**NBR**								
1	75.1	75.2	89.4	0.965	85.2	85.3	82.6	0.840
2,3	61.1	56.6	49.0	0.002	68.8	64.8	60.7	0.018
Unknown	70.9	72.8	70.7	0.563	79.5	79.3	77.0	0.807
**ER status**								
Negative	56.9	64.1	52.3	0.001	72.6	69.9	62.0	0.001
Positive	69.0	65.5	69.8	0.676	79.5	74.8	78.5	0.389
Unknown	66.1	70.6	62.7	0.538	75.3	83.1	71.1	0.386
**HER2 status**								
Negative	66.9	69.2	56.2	0.003	78.9	75.3	72.3	0.075
Positive	64.9	53.0	51.9	0.081	69.8	67.4	62.9	0.384
Unknown	67.9	64.8	65.8	0.507	74.7	72.6	68.9	0.188

**Table 5:** Ten-year Disease Free Survival rate and Overall Survival rate of breast cancer among breast cancer subtypes (N=2758)

	Disease free survival (%)						Overall survival (%)					
	All		Age ≤ 50		Age > 50		All		Age ≤ 50		Age > 50	
BMI	≥ 27	< 27	≥ 27	< 27	≥ 27	< 27	≥ 27	< 27	≥ 27	< 27	≥ 27	< 27
HR+/HER2-	52.9	55.9	45.0	57.2	51.7	61.7	56.3	73.4	71.4	77.1	44.4	66.6
*P* value	0.507		0.304		0.875		0.414		0.616		0.875	
HER2+	48.0	58.7	37.4	58.3	59.7	59.8	53.0	57.0	52.5	61.1	55.2	49.0
*P* value	0.204		0.029		0.810		0.211		0.265		0.643	
TNBC	42.6	56.4	37.2	57.8	43.3	54.4	56.3	68.3	56.6	72.9	59.9	61.0
*P* value	0.010		<0.001		0.732		0.009		<0.001		0.845	

*HR: hormone receptor; HER2: human epidermal growth factor receptor 2; TNBC: triple negative breast cancer.
